# Systemic Haemophilus parainfluenzae Infection Manifesting With Endocarditis and Membranoproliferative Glomerulonephritis

**DOI:** 10.7759/cureus.41086

**Published:** 2023-06-28

**Authors:** Sumbal Wajid, Larabe Farrukh, Lisa Rosenberg, Marium Faiz, Gurpreet Singh

**Affiliations:** 1 Internal Medicine, Albany Medical Center, Albany, USA

**Keywords:** haemophilus parainfluenzae endocarditis, endocarditis-associated glomerulonephritis, infectious endocarditis, infection related glomerulonephritis, membranoproliferative glomerulonephritis (mpgn), hacek

## Abstract

Infective endocarditis (IE) is a potentially fatal disease that is primarily caused by *Staphylococci* and *Streptococci*. The HACEK group of bacteria (*Hemophilus* *species*, *Aggregatibacter species*, *Cardiobacterium hominis*, *Eikenella corrodens, Kingella kingae*) account for only 1-3% of reported IE cases. IE has long been known to cause glomerulonephritis. The most common histologic patterns seen are crescentic and diffuse proliferative glomerulonephritis. Notably, membranoproliferative glomerulonephritis (MPGN) is one of the less common patterns seen with IE. We present a rare case of MPGN associated with *Haemophilus parainfluenzae *endocarditis.

A 56-year-old male with no significant past medical history presented to a local hospital with complaints of fever, night sweats, dyspnea, diarrhea, and dark urine for about a month. He was found to have a hemoglobin of 4g/dL, requiring multiple transfusions. He also had bilateral pleural effusions and pulmonary edema. In the following days, he had worsening renal function and was transferred to our hospital for further workup.

Initial labs showed anemia, thrombocytopenia, and leukocytosis. He had creatinine elevated at 5.28 mg/dL and a low estimated glomerular filtration rate (eGFR) of 12 mL/min/1.73m^2^. Urinalysis showed proteinuria, urine hemoglobin, urine white blood cells (WBCs), and red blood cells (RBCs). Blood cultures revealed *H.* *parainfluenzae*. Transesophageal echocardiogram (TEE) showed large vegetations with perforation of the mitral valve leaflet. Serology showed low complement levels. Renal biopsy displayed a membranoproliferative pattern of glomerulonephritis on light microscopy. The hepatitis panel was negative, as was the autoimmune workup. The patient was diagnosed with MPGN associated with *H.* *parainfluenzae* endocarditis. His complex clinical course required mitral valve replacement and aortic valve repair. He completed the course of antibiotics, with improvement in renal and cardiac function.

## Introduction

Infective endocarditis (IE) is a multi-system disease predominantly caused by *Streptococci *and *Staphylococci *(representing almost 75% of all cases) [1]. Many other microorganisms, including the HACEK group of bacteria (*Hemophilus species*, *Aggregatibacter species*, *Cardiobacterium hominis*, *Eikenella corrodens*, *Kingella kingae*), have also been identified to cause IE. HACEK organisms are responsible for only 1-3% of all cases of endocarditis [2]. Incidence of endocarditis with *Haemophilus parainfluenzae* is rare and accounts for only 0.8-1.3% of the cases. This group usually has an insidious course of disease onset, with a mean diagnostic delay of 1-3 months [2].

IE can involve kidneys, among other organs. Glomerular lesions associated with subacute IE were first described by Baehr G in 1912 [3]. The usual histologic patterns seen with IE include crescentic and diffuse proliferative glomerulonephritis [4]. Notably, membranoproliferative glomerulonephritis (MPGN) has rarely been reported with IE [4].

## Case presentation

The patient is a 56-year-old male with no significant past medical history who presented with complaints of fever, night sweats, dyspnea, diarrhea, and dark urine for the past one month to a local hospital, where he was found to have a hemoglobin of 4 g/dL (normal: 13.6-16.7), requiring multiple transfusions. Computed tomography (CT) scan of the chest showed bilateral pleural effusions. A diagnostic thoracentesis was performed, and pleural fluid analysis was consistent with transudative effusions. He had a transthoracic echocardiogram (TTE) that showed severe mitral regurgitation. Stool studies were negative for any infectious diarrhea. Endoscopy and colonoscopy were unremarkable for any source of bleeding. The patient developed worsening renal failure within a few days and was transferred to our hospital for further management.

On presentation, he was afebrile, tachycardic with a heart rate of 114 (normal: 60-90 bpm) and tachypneic with a respiratory rate of 25 (normal: 12-20/min), requiring supplemental oxygen through a high-flow nasal cannula to maintain oxygen saturations > 92%. His blood pressure was 103/70 mmHg. His physical examination revealed diminished breath sounds in the lower lung fields bilaterally, with accompanying pitting ankle edema. Initial labs showed anemia, thrombocytopenia, and leukocytosis. He had an elevated creatinine and low estimated glomerular filtration rate (eGFR) as indicated in Table 1. Urinalysis showed proteinuria, hemoglobinuria, and a large number of urine white blood cells (WBCs) and red blood cells (RBCs) (Table 2). Additional workup, including an iron panel, blood cultures, and serology, was sent. In the meanwhile, the patient was started on diuretics and broad-spectrum antibiotics with piperacillin-tazobactam and linezolid. Later, he developed an altered mental status. This was deemed secondary to uremia, and he was placed on renal replacement therapy.

**Table 1:** Laboratory findings

Test	Day 1	Day 2	Day 3	Reference Range
Hemoglobin	8.3	7.0	6.2	13.6-16.7 g/dL
White blood cell count	29.1	35.9	24.4	4-9 x 103/uL
Platelets	209	154	77	130-350 x 103/uL
Blood urea nitrogen	89	125	163	7-22 mg/dL
Creatinine	3.12	4.18	5.28	0.80-1.40 mg/dL
Estimated glomerular filtration rate	22	16	12	>60 mL/min/1.73m^2^

**Table 2:** Urine analysis WBCs: white blood cells, RBCs: red blood cells

Parameters	Results	Reference Values
Urine protein	3+	neg
Urine leukocyte esterase	2+	neg
Urine nitrites	neg	neg
Urine hemoglobin	3+	neg
Urine WBCs	20-50	0-5/HPF
Urine RBCs	50-100	0-2/HPF

Iron studies showed total iron 71 ug/dL (normal: 35-180), % iron saturation 33% (normal: 20-50), and ferritin 747 ng/mL (normal: 24-336). Serum total bilirubin was in the normal range, along with normal lactate dehydrogenase (LDH) and haptoglobin. Serology showed low complements levels and elevated titers for rheumatoid factor. In the setting of active urinary sediment and worsening renal function, a renal core biopsy was performed that showed immune-mediated MPGN with strong C3 and IgM positivity. Other possible etiologies for immune-mediated MPGN include hepatitis B/C, monoclonal gammopathies, and autoimmune diseases including systemic lupus erythematosus (SLE), Sjogren's syndrome or rheumatoid arthritis (RA), which were ruled out with negative viral panel, normal serum and urine protein electrophoresis and autoimmune workup with negative anti-nuclear antibody (ANA), anti-double stranded DNA (anti-dsDNA) at 12 IU/mL (normal: <30) and anti-CCP at 8 IU/mL (normal: <20). Blood cultures revealed *H. parainfluenzae*. His antibiotics were narrowed to ceftriaxone, following blood culture reports.

**Figure 1:**
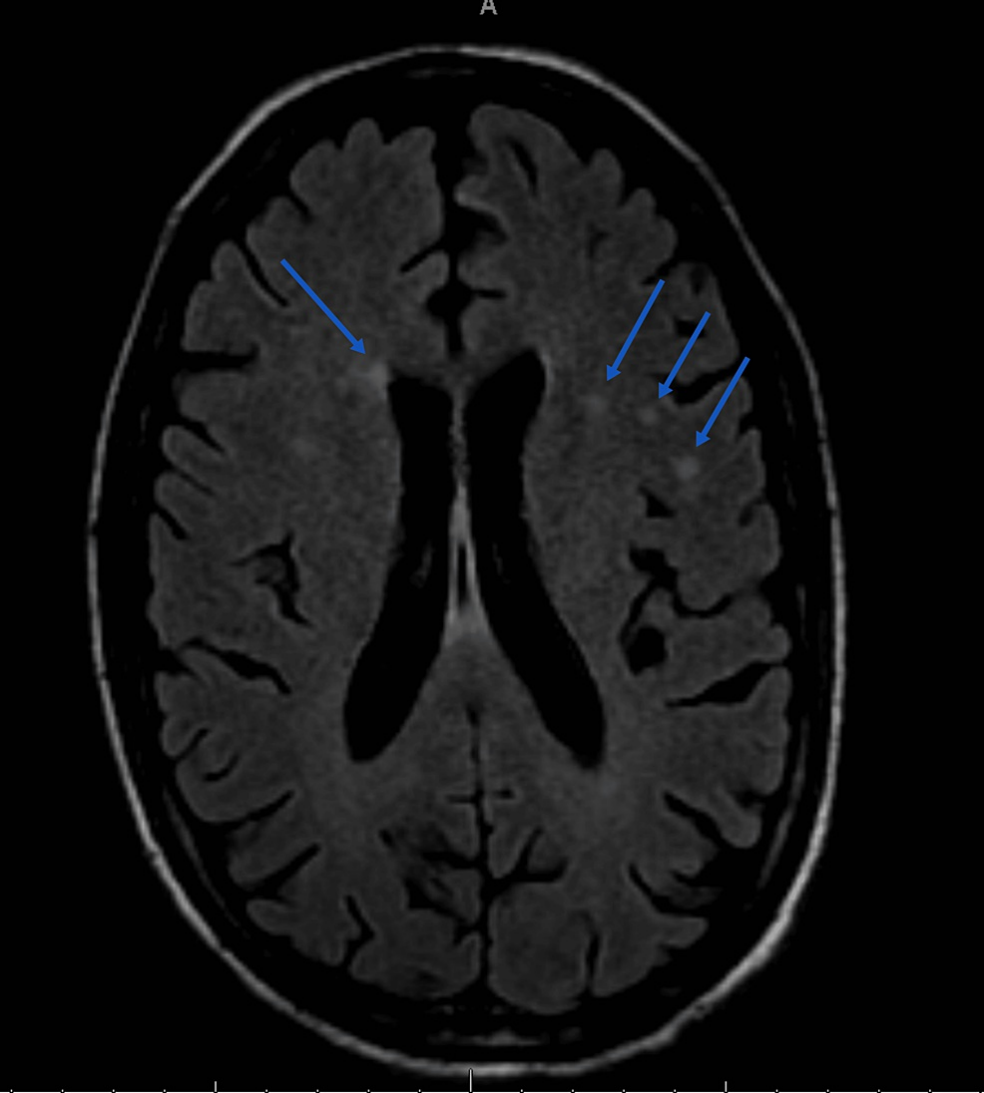
MRI brain with arrows pointing to restricted diffusion in the region of corona radiata and centrum semi-ovale. MRI: magnetic resonance imaging

The patient continued to have significant peripheral and pulmonary edema, despite diuretics and renal replacement therapy. A TTE was done, which showed mobile echo densities on the mitral valve suggestive of vegetations with mild to moderate regurgitation and left ventricular ejection fraction (LVEF) > 55%. Due to concerns for IE, a magnetic resonance imaging (MRI) scan of the brain (Figure 1) was done that showed few septic emboli in the corona radiata and centrum semi-ovale. A diagnosis of *H. parainfluenzae* endocarditis was made. Ceftriaxone and renal replacement therapy were continued, with continued improvement in his mental status.

**Figure 2:**
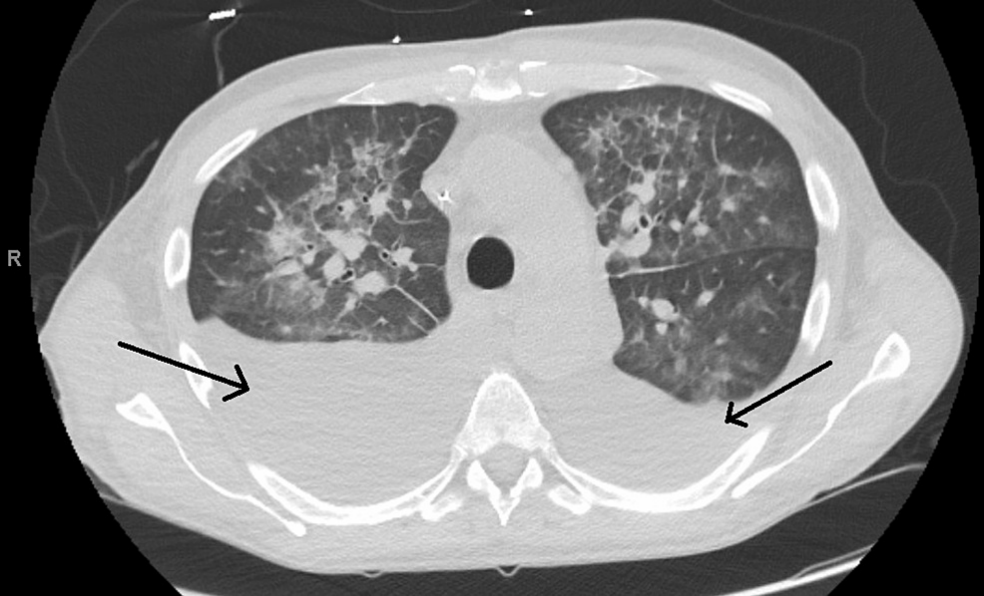
CT scan of the chest showing bilateral pleural effusions. CT: computed tomography

**Figure 3:**
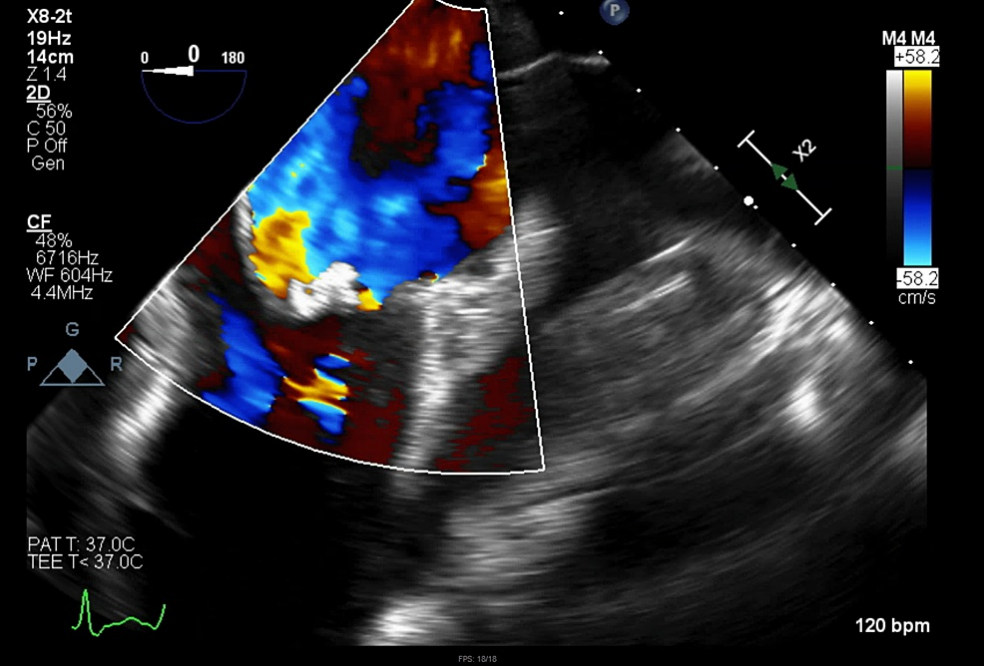
Transesophageal echocardiogram (TEE) showing mitral regurgitation.

**Figure 4:**
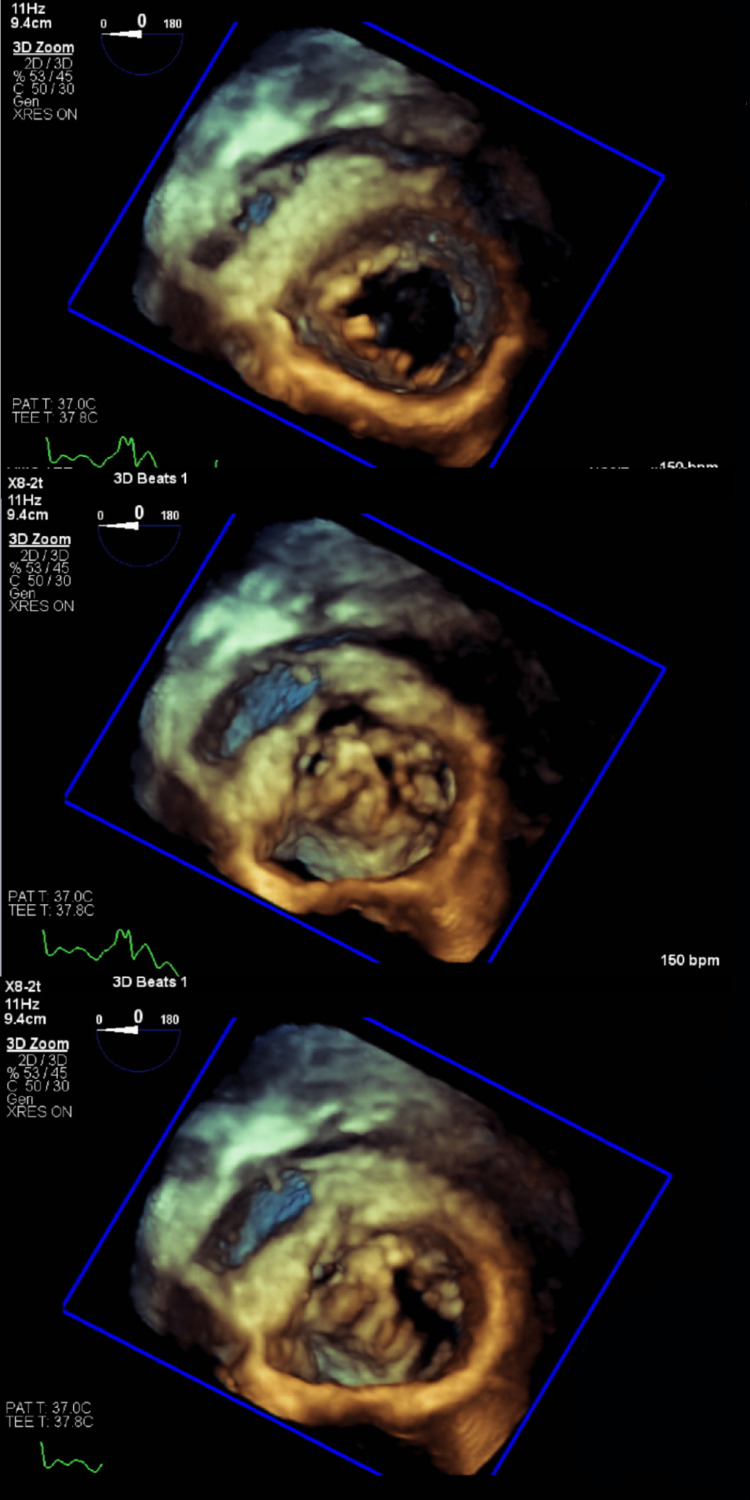
Transesophageal echocardiogram (TEE) showing mitral valve vegetations.

Due to persistent dyspnea despite increasing doses of diuretics, a limited follow-up TTE was done which confirmed worsening mitral regurgitation with LVEF > 55%. A repeat CT scan of the chest (Figure 2) showed large bilateral pleural fluid collections. Bilateral thoracentesis was done, and chest tubes were placed for pleural fluid drainage. At that point, a TEE showed severe mitral regurgitation associated with large mitral valve vegetations with perforation of the posterior leaflet (Figures 3-4). Cardiothoracic surgery was consulted for valve repair vs replacement. During surgery, a perforation was visualized on the posterior leaflet of the mitral valve with an extension of the infection into the anterior leaflet. The involved portions of leaflets were excised and replaced with a #27-sized Saint Jude mechanical mitral valve. He also underwent complex perforation repair on the non-coronary leaflet of the aortic valve, along with excision of the left atrial appendage.

He had initial improvement. However, later, the postoperative course was complicated by the development of cardiac tamponade with worsening LVEF of 30-35% noted on a follow-up TTE done two days after the initial surgery. He was taken back to the operating room for the pericardial window to drain the pericardial effusion. However, a clot was found in the right atrium, leading to the decision to do a full redo-sternotomy to evacuate the right atrial thrombus. The patient completed his antibiotic course and gradually improved over a few weeks. He was weaned off renal replacement therapy. A TTE performed three days following the procedure showed improved ejection fraction (35-39%) and resolution of pericardial effusion. His laboratory findings on the day of discharge are depicted in Table 3. The patient was started on goal-directed medical therapy for heart failure with beta-blocker (metoprolol succinate) and angiotensin-converting-enzyme (ACE) inhibitor (lisinopril). He was discharged with a follow-up with cardiology as an outpatient.

**Table 3:** Laboratory findings on the day of discharge

Test	Day of Discharge	Reference Range
Hemoglobin	10.2	13.6-16.7 g/dL
White blood cell count	4.5	4-9 x 103/uL
Platelets	194	130-350 x 103/uL
Blood urea nitrogen	12	7-22 mg/dL
Creatinine	0.80	0.80-1.40 mg/dL
Estimated glomerular filtration rate	124	>60 mL/min/1.73m^2^

## Discussion

IE is a serious and potentially life-threatening disease that can involve multiple systems of the human body, including but not limited to the heart, kidneys, brain, and skin. Although most cases are associated with *Staphylococci* and *Streptococci* [1], other microorganisms, including the HACEK group, have also been identified. The HACEK group of bacteria are fastidious gram-negative bacteria, which are part of the normal microbiota of the oral and upper respiratory tract in humans and mostly affect patients with underlying heart disease or prosthetic heart valves. This group usually has an insidious course of disease onset, with a mean diagnostic delay of 1-3 months [2]. In this case, despite no prior history of heart disease, blood cultures grew *H. parainfluenzae*, and TEE showed large vegetations on his mitral valve leaflets, confirming the diagnosis of *H. parainfluenzae*-related endocarditis.

Renal manifestations of IE depend on the severity of the underlying infection and the degree of kidney involvement. The kidneys are particularly vulnerable to damage during IE because they receive a large amount of blood flow and are responsible for filtering waste products from the blood. Glomerulonephritis is a common clinical feature of IE. The pathophysiology involves the activation of the immune system to fight off the infection that can cause inflammation and deposition of immune complexes in glomeruli leading to GN [5]. The most common presenting symptom is hematuria, with others being hypertension, proteinuria, and peripheral edema.

Diagnosis of GN typically involves a combination of blood tests, urine tests, imaging, and kidney biopsy. Abnormalities in serological testing usually seen with IE-related GN include hypocomplementemia, high titers of rheumatoid factor, and type III cryoglobulinemia [6]. The most common histologic patterns seen on biopsy are necrotizing and crescentic GN, followed by diffuse proliferative GN. Other patterns seen include focal proliferative GN and mild mesangial proliferative GN [6]. Notably, MPGN is the least commonly reported pattern seen in GN related to infection [4]. Currently, only one case of MPGN associated with subacute endocarditis has been reported in the literature [7]. Our case developed acute uremic encephalopathy during his hospitalization and was found to have low complement levels. Interestingly, renal biopsy showed immune-mediated MPGN with immunofluorescence showing strong C3 and IgM positivity. This was an unusual finding, as MPGN is a rare glomerular lesion with IE-related GN.

Treatment of endocarditis complicated by MPGN typically involves antibiotics to treat the underlying infection. The indications for surgery include acute heart failure related to aortic/mitral regurgitation, uncontrolled infection, prevention of embolism, and isolated very large vegetation.

## Conclusions

We present a rare case of *H. parainfluenzae* endocarditis associated with MPGN in a patient requiring complex medical care involving multiple specialties. The treatment of endocarditis associated with MPGN involves addressing both the infection of the heart and the underlying kidney disease. The primary treatment option is the administration of antibiotics. In some cases, surgical intervention may be necessary to remove infected tissue or repair damaged heart valves. The decision to use immunosuppressive therapy to reduce kidney inflammation should be carefully considered, as it may increase the risk of infection. It is important to note that the treatment approach for endocarditis associated with MPGN may vary depending on the individual patient's condition and the recommendations of the treating healthcare team. The management should be carried out by a multidisciplinary team involving cardiologists, nephrologists, and infectious disease specialists to ensure comprehensive care.
